# Acute Footshock Stress Induces Time-Dependent Modifications of AMPA/NMDA Protein Expression and AMPA Phosphorylation

**DOI:** 10.1155/2016/7267865

**Published:** 2016-02-04

**Authors:** Daniela Bonini, Cristina Mora, Paolo Tornese, Nathalie Sala, Alice Filippini, Luca La Via, Marco Milanese, Stefano Calza, Gianbattista Bonanno, Giorgio Racagni, Massimo Gennarelli, Maurizio Popoli, Laura Musazzi, Alessandro Barbon

**Affiliations:** ^1^Biology and Genetic Division, Department of Molecular and Translational Medicine, University of Brescia, 25123 Brescia, Italy; ^2^Laboratorio di Neuropsicofarmacologia e Neurogenomica Funzionale, Dipartimento di Scienze Farmacologiche e Biomolecolari and CEND, Università degli Studi di Milano, 20133 Milano, Italy; ^3^Department of Pharmacy, Unit of Pharmacology and Toxicology and Center of Excellence for Biomedical Research, University of Genoa, 16148 Genoa, Italy; ^4^Unit of Biostatistics and Biomathematics, Department of Molecular and Translational Medicine, University of Brescia, Brescia, Italy; ^5^Genetic Unit, IRCCS Centro San Giovanni di Dio Fatebenefratelli, 25125 Brescia, Italy

## Abstract

Clinical studies on patients with stress-related neuropsychiatric disorders reported functional and morphological changes in brain areas where glutamatergic transmission is predominant, including frontal and prefrontal areas. In line with this evidence, several preclinical works suggest that glutamate receptors are targets of both rapid and long-lasting effects of stress. Here we found that acute footshock- (FS-) stress, although inducing no transcriptional and RNA editing alterations of ionotropic AMPA and NMDA glutamate receptor subunits, rapidly and transiently modulates their protein expression, phosphorylation, and localization at postsynaptic spines in prefrontal and frontal cortex. In total extract, FS-stress increased the phosphorylation levels of GluA1 AMPA subunit at Ser^845^ immediately after stress and of GluA2 Ser^880^ 2 h after start of stress. At postsynaptic spines, stress induced a rapid decrease of GluA2 expression, together with an increase of its phosphorylation at Ser^880^, suggesting internalization of GluA2 AMPA containing receptors. GluN1 and GluN2A NMDA receptor subunits were found markedly upregulated in postsynaptic spines, 2 h after start of stress. These results suggest selected time-dependent changes in glutamatergic receptor subunits induced by acute stress, which may suggest early and transient enhancement of AMPA-mediated currents, followed by a transient activation of NMDA receptors.

## 1. Introduction

Stress can be defined as any condition that perturbs the physiological homeostasis [1]. A stressful event rapidly activates both the hypothalamic-pituitary-adrenocortical axis, leading to secretion of glucocorticoids (mainly cortisol in humans, corticosterone in rats), and the autonomic nervous system, which releases catecholamines (noradrenaline, adrenaline). The stress response is physiologically proadaptive, when efficiently turned on and then shut off, but may became maladaptive, particularly in subjects with a genetic background of vulnerability or when the stressful stimulus is chronic or overwhelming [2, 3].

The prefrontal cortex (PFC), a region involved in working memory, decision-making, and behavioral flexibility, as well as in social interaction and emotional processing, is a main target of the stress hormones [4–6]. A large body of literature has consistently shown that the fast response to stress involves increased attention, vigilance, and improved PFC-mediated cognitive performance, mainly mediated by potentiation of glutamate transmission [7–9]. Indeed, acute stress and glucocorticoids rapidly modulate glutamate release and excitatory synaptic transmission in PFC [8, 10–12]. In particular, it has been shown that acute stress induces a rapid and transient enhancement of N-methyl-D-aspartic acid- (NMDA-) and *α*-amino-3-hydroxy-5-methyl-4-isoxazolepropionic acid- (AMPA-) receptor-mediated currents in PFC in juvenile rats, together with increasing the surface expression of AMPA and NMDA receptor subunits [10, 11].

Taken together, these data strongly suggest that the enrichment, localization, and posttranslational modifications, as well as posttranscriptional and translational regulations of glutamate receptors, may be involved in the neuronal response to behavioral stress.

In this study, we exposed adult male rats to acute footshock- (FS-) stress and investigated time-dependent modifications of AMPA and NMDA receptor subunits mRNA and protein expression, RNA editing, and posttranslational regulation. The analyses have been performed in the prefrontal and frontal cortex (PFC/FC) at different time points (immediately after the 40 min of stress and 2 hours and 24 hours after stress start), to monitor the early and delayed effects of acute stress on the regulatory mechanisms of ionotropic glutamate receptors.

The results provided here indicate that exposure to acute stress causes transient and time-dependent subunit-specific changes in glutamate receptor, in line with previously observed adaptive modifications of excitatory synaptic transmission in the PFC/FC.

## 2. Materials and Methods

### 2.1. Footshock Stress Procedure

All experimental procedures involving animals were performed in accordance with the European Community Council Directive 86/609/EEC and were approved by the Italian legislation on animal experimentation (Decreto Ministeriale 116/1992). Experiments were performed with adult male Sprague-Dawley rats (275–300 g). Rats were housed two per cage and maintained on a 12/12 h light/dark schedule (lights on at 7:00 am), in a temperature controlled facility with free access to food and water. The experiments were performed during the light phase (between 9:00 and 12:00 am), at least one week after arrival from the supplier (Charles River, Wilmington, MA, USA). The footshock- (FS-) stress protocol was performed essentially as previously reported (40 min FS-stress: 0.8 mA, 20 min total of actual shock with random intershock length between 2 and 8 sec) [8, 12, 13]. Sham-stressed rats (controls) were kept in the stress apparatus without delivering of shocks. Rats were killed by decapitation at different time points (10 rats/group): immediately after the stress session (*t* = 0) and 2 or 24 h after stress start. The 2 and 24 h groups were left undisturbed in their cages after the 40 min stress session. Sham-groups were prepared at each time point, as specific controls for respective stressed groups.

The whole frontal lobe, referred to as PFC/FC, was quickly dissected on ice and right and left hemiareas were randomly assigned to RNA extraction or postsynaptic spine membranes (triton insoluble fraction; TIF) purification.

Serum corticosterone levels were measured using a commercial kit (Corticosterone ELISA kit, Enzo Life Sciences, Farmingdale, NY, USA).

### 2.2. RNA Extraction and Retrotranscription

Samples from PFC/FC of each animal were homogenized, and total RNA was extracted using TRIZOL reagent (Life Technologies, Milano, Italy). RNA was recovered by precipitation with isopropyl alcohol, washed with a 75% ethanol solution, and dissolved in RNase-free water. RNA quantification and quality controls were carried out using both spectrophotometric analysis and AGILENT Bioanalyzer 2100 lab-on-a-chip technology (AGILENT Technologies, Santa Clara, CA, USA). Reverse-transcription (RT) was done using Moloney murine leukemia virus-reverse transcriptase (MMLV-RT) (Life Technologies). Briefly, 2.5 *μ*g of total RNA from each sample was mixed with 2.2 *μ*L of 0.2 ng/*μ*L random hexamers, 10 *μ*L of 5x buffer, 10 *μ*L of 2 mM dNTPs, 1 *μ*L of 1 mM DTT, 0.4 *μ*L of 33 U/*μ*L RNaseout, and 2 *μ*L MMLV-RT (200 U/*μ*L) in a final volume of 50 *μ*L. The reaction mix was incubated at 37°C for 2 h and the enzyme was then heat inactivated at 95° for 10 min.

### 2.3. Quantitative Real-Time PCR and RNA Editing Quantification

RNA expression pattern of the glutamate receptors was analyzed by means of an Applied Biosystems 7500 Real-Time PCR system (Applied Biosystems, Foster City, CA, USA). PCR was carried out by using TaqMan Universal PCR Master Mix (Applied Biosystems). 25 ng of sample was used in each real-time PCR reaction (TaqMan Gene Expression Assay id probes: GluA1: Rn00709588_m1; GluA2: Rn00568514_m1; GluN1: RN01436038_m1; GluN2A: Rn00561341_m1; GluN2B: Rn00561352_m1, Applied Biosystems). The expression ratio of target genes in treated sample groups, compared to control group, was calculated using the ΔΔCt method and H2AFZ, GAPDH, and PolII geometric mean as reference (ID H2AFZ TaqMan probe: Rn00821133_g1; ID GAPDH: Rn99999916_m1; ID PolII: Rn00580118_m1). Each individual determination was repeated in triplicate. The quantification for AMPA receptor subunits GluA2 Q/R and GluA2, GluA3, and GluA4 R/G editing levels were measured by sequence analysis as previously described [14, 15].

### 2.4. Protein Extracts and Western Blotting

PFC/FC were homogenized in 0.32 M ice-cold sucrose containing 1 mM HEPES, 1 mM MgCl_2_, 1 mM EDTA, 1 mM NaHCO_3_, and 0.1 mM PMSF, at pH 7.4, 2 mg/mL of protease inhibitor cocktail (Thermo scientific, Rockford, IL, USA), and phosphatases inhibitors (Sigma-Aldrich, Milan, Italy), pH 7.4. 200 *μ*L of homogenate was aliquoted and immediately frozen.

Triton-X-100 insoluble fractions (TIF) were purified as previously reported [12]. The homogenized tissue was centrifuged at 1000 ×g for 10 min. The resulting supernatant (S1) was centrifuged at 3000 ×g for 15 min to obtain a crude membrane fraction (P2 fraction). The pellet was resuspended in 1 mM HEPES and centrifuged at 100,000 ×g for 1 h. The pellet (P3) was resuspended in buffer containing 75 mM KCl and 1% Triton-X-100 and centrifuged at 100,000 ×g for 1 h. The supernatant was stored and referred to as Triton-X-100-soluble fraction (TSF) (S4). The final pellet (P4) was homogenized in 20 mM HEPES. Then, an equal volume of glycerol was added, and this fraction, referred to as TIF, was stored at −80°C until processing.

The BCA protein concentration assay (Sigma-Aldrich, St. Louis, MO, USA) was used for protein quantitation. Before electrophoresis, each sample was incubated at 75°C for 10 min. Equal amounts of proteins were applied to precast SDS polyacrylamide gels (4–12% NuPAGEBis-Tris gels; Life Technologies, Milan, Italy), and proteins were electrophoretically transferred to a Hybond-P PVDF Transfer Membrane (GE Healthcare Life Science), for 2 h at a 1 mA/cm^2^ of membrane surface. Membranes were blocked for 60 min with 3–5% nonfat dry milk or 5% BSA in TBS-T (Tris-buffered saline with 0.2% Tween-20, Sigma-Aldrich, Milan, Italy). Immunoblotting was carried out overnight at 4°C with specific antibodies against phosphoSer^831^-GluA1 (1 : 1000, cod. ab109464, Abcam, Cambridge, UK), phosphoSer^845^-GluA1 (1 : 1000, cod. ab3901, Abcam), and phosphoSer^880^-GluA2 (1 : 1.000, cod. Ab52180, Abcam). Immunoblotting was also carried out on the same stripped membranes with antibodies against total GluA1 (1 : 200, cod. AGC004, Alomone Labs, Jerusalem, Israel) and GluA2 (1 : 2500, cod. AGC005, Alomone) in blocking buffer. Primary antibodies were used to detect GluN1 (1 : 500, cod. AB9864, Millipore, Billerica, MA, USA), GluN2A (1 : 500, AB1555P, Millipore), and GluN2B (1 : 500, cod. 454582, Calbiochem-Millipore). Mouse monoclonal anti-GAPDH (1 : 40.000, cod. Mab374, Millipore) or rabbit monoclonal anti-*β*-Actin (1 : 3000, cod.04-1116, Millipore) were used as internal controls. Membranes were washed five times with TBS-Tween-20 0.2% and incubated for 1 h at room temperature with AP-conjugated secondary antibodies (Promega, Milan, Italy). Immunolabeled proteins were detected by incubation with Supersignal West Pico Chemiluminescent Substrate (Pierce, Rockford, IL, USA) or CDPStar (Roche Applied Science) detection reagents and then exposed to imaging film. Prestained Novex Sharp Protein Standards (Life Technologies) were used as molecular weight standards loaded on the same gel. The intensity of immunoreactive bands was analyzed with Image-Pro Plus. Data are presented as optical density ratios of the investigated protein band normalized for GAPDH or *β*-Act bands in the same line and are expressed as percentage of controls. The levels of GluA phosphorylated subunits were normalized to total GluA levels, based on previous reports [16, 17].

### 2.5. Data Analysis

All the analyses were carried out in individual animals (independent determinations).

Preliminary data inspection showed a fairly constant coefficient of variation among groups, as well as a multiplicative effect on the mean. Therefore, we modeled data using a gamma regression model with log-link (via Generalized Linear Models, GLM [18]), with treatment (stress, control), time, and their interaction as predictors. Where needed, a robust Generalized Linear Model was used to account for potential outliers. Due to some additional heteroskedasticity in corticosterone levels between groups, tests were performed using “sandwich” robust standard error estimates. Data in the text are reported as estimated fold changes (FC) and 95% confidence intervals (CI 95%). The interaction between treatment and time (treatment-×-time) was considered the main effect of interest, as it indicates a differential effect on stressed versus control groups during time. Pairwise contrasts *p* values between groups were adjusted by Bonferroni Post Hoc Test (reported as *p*_adj_). Statistical significance was assumed at *p* < 0.05.

For simplicity, data on graphs are represented as estimated group mean values + standard errors of the means (SEM). Stressed groups are represented as percentage of controls at each time point. Statistical analysis was carried out by using *R* [19].

## 3. Results

### 3.1. Corticosterone Levels

To test the efficacy of the stress protocol, we evaluated plasma corticosterone levels in all the animals. As expected, the FS-stress procedure markedly and transiently increased serum corticosterone levels as shown in Table 1.

We observed a significant increase in corticosterone levels in stressed animals sacrificed immediately after the stress session (*t* = 0  FC = 5.11, CI 95% = 2.42–10.77, *p*_adj_ < 0.001), with a relatively slow decrease in the following time points (2 h FC = 4.83, CI 95% = 2.19–10.64, *p*_adj_ < 0.001; 24 h FC = 2.49, CI 95% = 0.84–7.39, *p*_adj_ = 0.13).

### 3.2. Acute Stress Does Not Induce Any Alteration in Transcriptional Levels and Editing of Ionotropic Glutamate Receptors

mRNA expression analysis of glutamate receptor subunits showed no changes in transcript levels, at the three time points analyzed (see Supplemental Figure  1 in Supplementary Material available online at http://dx.doi.org/10.1155/2016/7267865). Furthermore, no alterations were observed for Q/R and R/G editing sites of GluA2, GluA3, and GluA4 AMPA subunits (Supplemental Figure  2).

### 3.3. Modulation of AMPA Receptor Subunits Expression and Phosphorylation Induced by Acute Stress

To assess time-dependent changes induced by acute stress in glutamate receptor subunits expression, Western blot analyses for AMPA and NMDA receptor subunits were performed on PFC/FC total homogenates and purified postsynaptic spine membranes (TIF) of rats subjected to acute FS-stress and sacrificed at the different time points.

**Figure 1:**
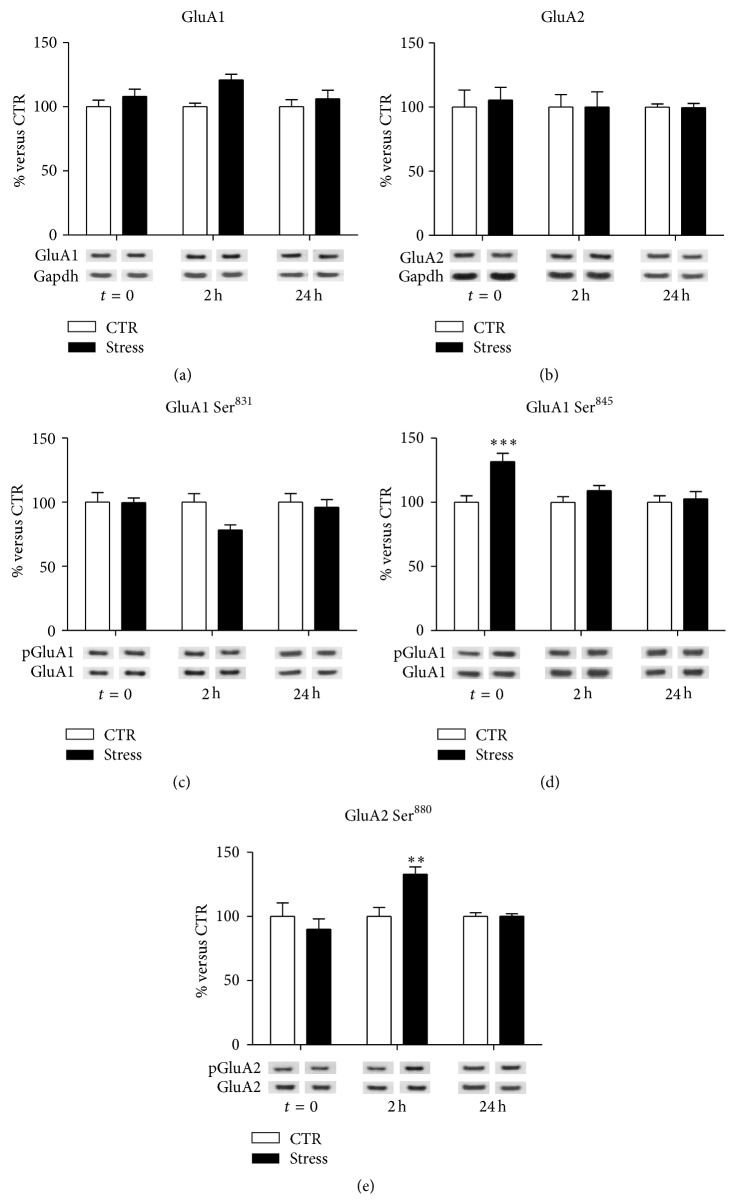
Time-dependent changes of protein expression levels of GluA1 (a), GluA2 (b), GluA1 phospho-Ser^831^ (c), GluA1 phospho-Ser^845^ (d), and GluA2 phospho-Ser^880^ (e) in PFC/FC total homogenate of rats subjected to FS-stress and sacrificed immediately after stress and 2 h and 24 h from stress beginning. Data are represented as percentage of controls at each time point, as means ± SEM (*n* = 8). Statistics: Generalized Linear Models (GLM) and Bonferroni Post Hoc Test (see Section 2 for details). ^*∗∗*^ *p* < 0.01; ^*∗∗∗*^ *p* < 0.001.

In total PFC/FC homogenate, no significant effects of FS-stress were found on the total expression of GluA1 and GluA2 AMPA receptor subunits at different time points (GluA1: interaction term, *p* = 0.47; GluA2: interaction term, *p* = 0.94) (Figures 1(a) and 1(b), resp.), although a trend for increase could be observed for GluA1, 2 h after the stress beginning (FC = 1.21, *p*_adj_ = 0.08).

No significant changes were also observed for GluA1 Ser^831^ phosphorylation in FS-stress animals at different time points (interaction term *p* = 0.15, Figure 1(c)), despite single comparison at 2 hours after stress had a marginally significant effect (FC = 0.78; *p* = 0.045). In contrast, we measured a significant treatment-×-time interaction for GluA1 Ser^845^ phosphorylation (*p* = 0.026, Figure 1(d)). In particular, exclusively immediately after the stress protocol, a marked upregulation of GluA1 Ser^845^ phosphorylation was observed (FC = 1.32, CI 95% = 1.12–1.55, *p*_adj_ < 0.001), with no significant variations at other time points (2 h FC = 1.09, CI 95% = 0.93–1.28, *p*_adj_ = 0.49; 24 h FC = 1.03, CI 95% = 0.87–1.21, *p*_adj_ = 0.97). Moreover, we found a significant treatment-×-time effect for GluA2 Ser^880^ phosphorylation (*p* = 0.002; Figure 1(e)): acute stress caused an increase in GluA2 Ser^880^ phosphorylation 2 h after its start (FC = 1.33, CI 95% = 1.09–1.62, *p*_adj_ = 0.0015), while no significant changes were observed at the other time points (2 h FC = 0.90, *p*_adj_ = 0.62; 24 h FC = 1.00, *p*_adj_ = 0.99).

**Figure 2:**
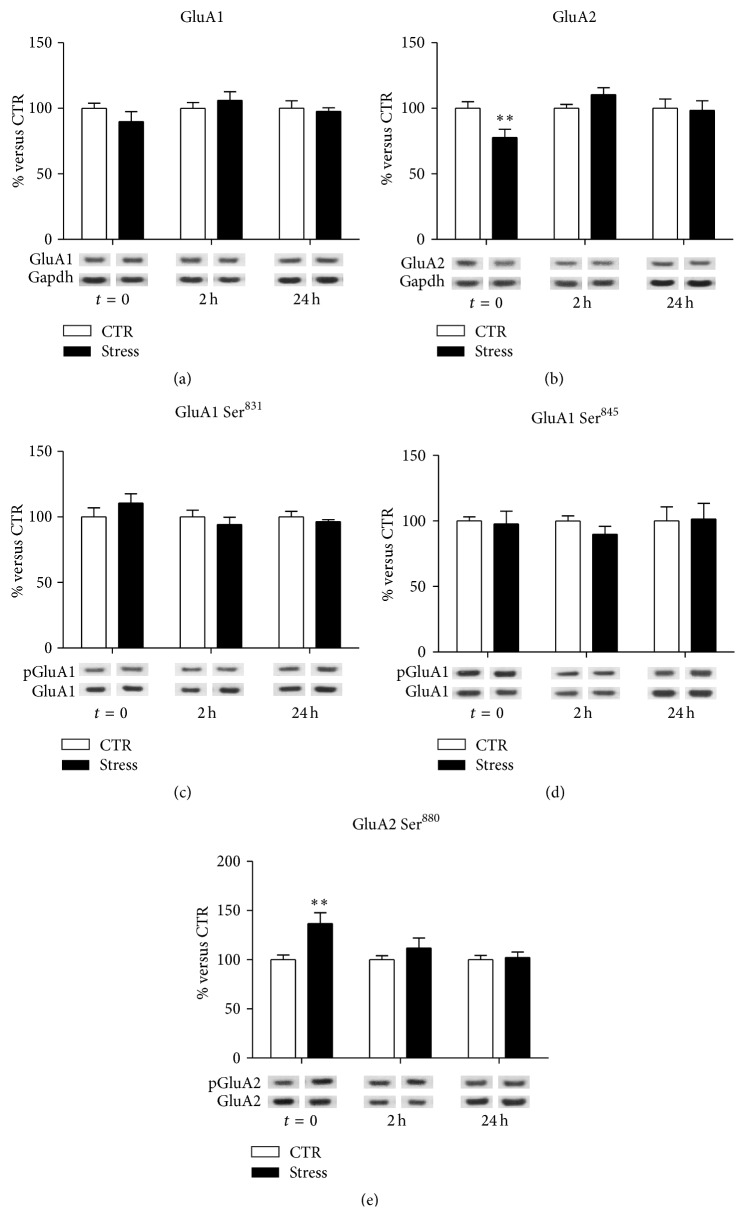
Time-dependent changes of protein expression levels of GluA1 (a), GluA2 (b), GluA1 phospho-Ser^831^ (c), GluA1 phospho-Ser^845^ (d), and GluA2 phospho-Ser^880^ (e) in PFC/FC postsynaptic spine membranes of rats subjected to FS-stress and sacrificed immediately after stress and 2 h and 24 h from stress beginning. Data are represented as percentage of controls at each time point, as means ± SEM (*n* = 8). Statistics: Generalized Linear Models (GLM) and Bonferroni Post Hoc Test (see Section 2 for details). ^*∗∗*^ *p* < 0.01.

At postsynaptic membranes, no significant modifications were observed for GluA1 (interaction term, *p* = 0.32; Figure 2(a)), while treatment-×-time interaction was significant for GluA2 subunit (*p* = 0.013; Figure 2(b)), with a significant downregulation immediately after the stress protocol (FC = 0.77, CI 95% = 0.63–0.95, *p*_adj_ = 0.01). No significant modifications were found in GluA1 phosphorylation at Ser^831^ (interaction term, *p* = 0.27; Figure 2(c)) or Ser^845^ (interaction term *p* = 0.76; Figure 2(d)). On the contrary, we observed a significant treatment-×-time interaction for GluA2 at Ser^880^ phosphorylation levels (*p* = 0.025; Figure 2(e)), which were significantly increased immediately after the stress protocol (FC = 1.37, CI 95% = 1.11–1.69, *p*_adj_ = 0.0013) and reduced at following time points (2 h FC = 1.12, CI 95% = 0.91–1.38, *p*_adj_ = 0.49; 24 h FC = 1.02, CI 95% = 0.83–1.26, *p*_adj_ = 0.99).

### 3.4. Acute Stress Induces Alterations in NMDA Receptor Subunits Expression

**Figure 3:**
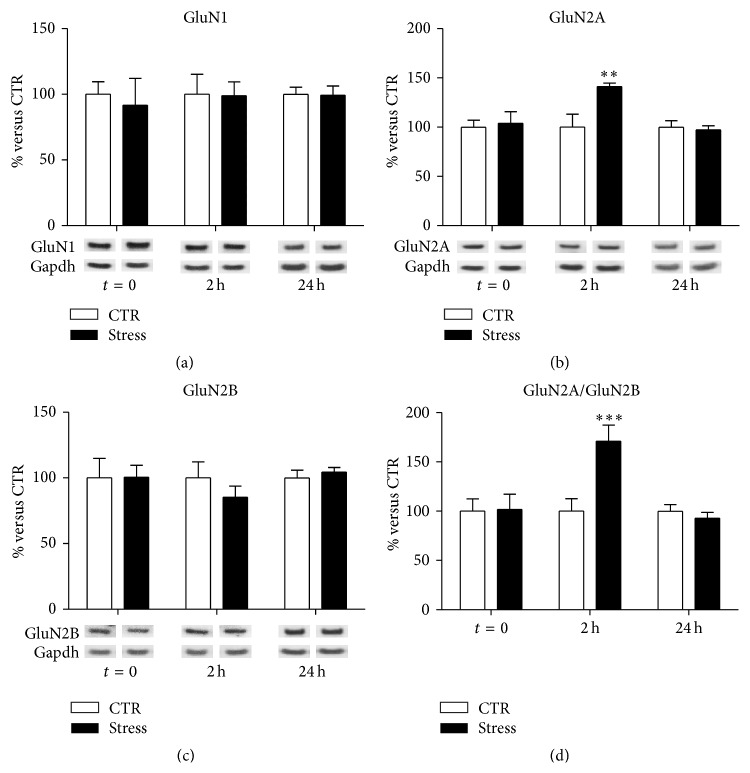
Time-dependent changes of protein expression levels of GluN1 (a), GluN2A (b), GluN2B (c), and GluN2A/GluN2B (d) in PFC/FC total homogenate of rats subjected to FS-stress and sacrificed immediately after stress and 2 h and 24 h from stress beginning. Data are represented as percentage of controls at each time point, as means ± SEM (*n* = 8). Statistics: Generalized Linear Models (GLM) and Bonferroni Post Hoc Test (see Section 2 for details). ^*∗∗*^ *p* < 0.01; ^*∗∗∗*^ *p* < 0.001.

In total PFC/FC homogenates, we found no effect of stress on GluN1 subunit expression levels at different time points (interaction term, *p* = 0.94, Figure 3(a)). Instead, with regard to GluN2A, a significant treatment-×-time interaction term was found (*p* = 0.022; Figure 3(b)). Indeed, total GluN2A expression levels were found increased in total homogenates of PFC/FC from FS-stress rats, selectively 2 h after the beginning of stress (FC = 1.41, CI 95% = 1.07–1.86, *p*_adj_ = 0.008), and not at the other time points analyzed (*t* = 0 FC = 1.04, *p*_adj_ = 0.99; 24 h FC = 0.97, *p*_adj_ = 0.99). With regard to GluN2B subunit, a trend for decrease although not statistically significant was observed 2 h after the beginning of stress (FC = 0.85, *p*_adj_ = 0.53; Figure 3(c)).

Given the key role of GluN2A/GluN2B ratio in regulating glutamatergic synapses activity [20], the ratio between the two subunits has also been calculated (Figure 3(d)). We found a significant treatment-×-time interaction effect (*p* = 0.003), with GluN2A/GluN2B ratio significantly higher in PFC/FC total homogenates from stressed rats sacrificed 2 h after the stress beginning (FC = 1.71, CI 95% = 1.22–2.40, *p*_adj_ < 0.001), and no significant changes in GluN2A/GluN2B ratio at other time points (*t* = 0 FC = 1.02, *p*_adj_ = 0.99; 24 h FC = 0.93, *p*_adj_ = 0.94).

**Figure 4:**
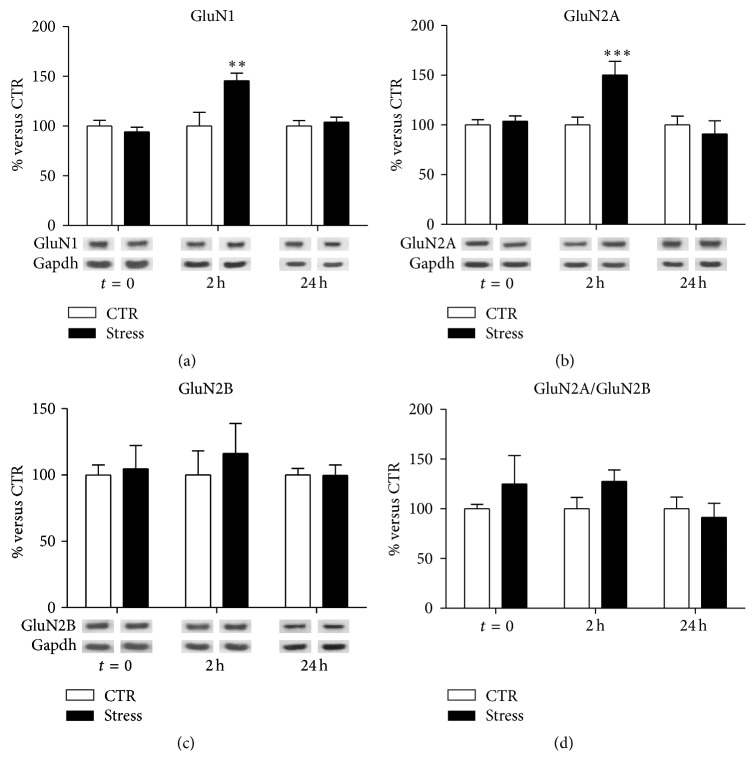
Time-dependent changes of protein expression levels of GluN1 (a), GluN2A (b), GluN2B (c), and GluN2A/GluN2B (d) in PFC/FC postsynaptic spine membranes of rats subjected to FS-stress and sacrificed immediately after stress and 2 h and 24 h from stress beginning. Data are represented as percentage of controls at each time point, as means ± SEM (*n* = 8). Statistics: Generalized Linear Models (GLM) and Bonferroni Post Hoc Test (see Section 2 for details). ^*∗∗*^ *p* < 0.01; ^*∗∗∗*^ *p* < 0.001.

At postsynaptic membranes, we observed a significant treatment-×-time interaction (Robust GLM, *p* = 0.005) for GluN1 expression levels (Figure 4(a)), which were significantly increased in FS-stressed rats sacrificed 2 h after the beginning of stress (FC = 1.36, CI 95% = 1.05–1.68, *p*_adj_ = 0.0013). A similar result was found for GluN2A subunit (Figure 4(b)). GluN2A protein expression level showed a significant stress-×-time interaction (Robust GLM, *p* = 0.0009), with a marked increase 2 h after stress beginning (FC = 1.50, CI 95% = 1.16–1.93, *p*_adj_ = 0.0005). On the contrary, no alterations were found either in postsynaptic level of GluN2B (interaction term, *p* = 0.85; Figure 4(c)) or in GluN2A/GluN2B ratio (interaction term, *p* = 0.39; Figure 4(d)).

## 4. Discussion

We report here that acute footshock- (FS-) stress, although inducing no transcriptional or posttranscriptional alterations of ionotropic AMPA and NMDA glutamate receptor subunits, modulates, in a time- and subunit-dependent way, their protein expression, phosphorylation, and localization at postsynaptic spines in PFC/FC of rats.

In particular, FS-stress rapidly increased phosphorylation of GluA1, selectively at Ser^845^ (not at Ser^831^), and of GluA2 at Ser^880^ in total homogenate, while reducing GluA2 levels, together with increasing its phosphorylation at Ser^880^, in postsynaptic spine membranes. Acute stress exerted no effect on GluA1 and GluA2 protein expression levels in total homogenate, as previously reported [10]. All the changes in AMPA receptor subunits expression and phosphorylation levels were selectively measured immediately after the 40 min of stress session (except for increased GluA2 phospho-Ser^880^ levels in total homogenate, which were selectively increased 2 h after stress start), suggesting fast and transient modulation of AMPA receptor subunits at PFC/FC synapses induced by acute stress.

Phosphorylation of GluA1 at Ser^831^ and Ser^845^ has been shown to modulate potentiation of AMPA receptor-mediated synaptic currents and to be involved in both Long Term Potentiation (LTP) and Long Term Depression (LTD) [21]. In particular, phosphorylation at Ser^845^ increases the open channel probability, and the peak amplitude of currents mediated by AMPA receptors [21]. Therefore, the increase of GluA1 phosphorylation at Ser^845^ rapidly induced by FS-stress is in line with increased AMPA receptor currents.

Phosphorylation of GluA2 at Ser^880^ was shown to affect its association with PDZ domain-containing proteins, thereby modifying trafficking and redistribution of the subunit at synaptic sites, facilitating GluA2 internalization [22–25], and subsequent lysosomal degradation [26]. GluA2 is a critical subunit in determining the function of AMPA receptors. Indeed, GluA2-containing AMPA receptors are Ca^2+^-impermeable and have a relatively low single channel conductance [27], while AMPA receptors lacking GluA2 subunit have a higher Ca^2+^ permeability and conductance [28, 29]. Intriguingly, it was shown that homomeric GluA1 AMPA receptors are delivered to synapses after LTP induction, whereas homomeric GluA2 or GluA3 AMPA receptors are constitutively inserted [30, 31].

Taken together, this body of evidence strongly suggests that acute FS-stress, increasing GluA1 phosphorylation at Ser^845^ and reducing the levels of GluA2-containing AMPA receptors at postsynaptic membranes, may rapidly and transiently activate AMPA receptor-mediated synaptic currents.

We have also found here that acute FS-stress markedly increased GluN2A expression levels and GluN2A/GluN2B ratio in PFC/FC homogenate and GluN1 and GluN2A protein levels in postsynaptic spine membranes, 2 h after the stress session. Notably, no changes were detected in NMDA receptor subunits at other time points, suggesting a time-dependent modulation of GluN2A and GluN2B subunits induced by acute stress.

In the forebrain, NMDA receptors are composed of one GluN1 subunit and one or more GluN2A or GluN2B subunits, and the precise combination of subunits determines the functional properties of the receptor [32]. It is well known that NMDA receptor subunit composition changes during development: while GluN2B is abundant in the early postnatal brain, the level of GluN2A, characterized by faster rising and decay kinetics, increases progressively during development [33]. In the adult brain, GluN2A is enriched at synaptic sites, while GluN2B is mainly extrasynaptic [34], and the GluN2A/GluN2B ratio was shown to be dependent on neuronal activity [35]. In particular, since increased GluN2A/GluN2B ratio is related with increased synaptic stimulation and transmission, its dynamic regulation is a major determinant of synaptic plasticity [36]. In this context, the increase of GluN2A/GluN2B ratio in homogenate, together with enrichment of GluN1- and GluN2A-containing NMDA receptors in postsynaptic spine membranes measured 2 h, but not 24 h, after the start of FS-stress, is in line with a delayed and transient enhancement of NMDA receptor-mediated synaptic currents.

In line with our results, in previous studies it was shown that both acute stress* in vivo* and short-term incubation of PFC neurons with corticosterone* in vitro* increase AMPA and NMDA receptor-mediated synaptic transmission and expression levels at membranes [10, 37]. However, contrary to the fast and transient effect measured after FS-stress, acute forced-swim stress was shown to induce a long-lasting increase (from 1–4 h, to 24 h after stress) of AMPA and NMDA-mediated excitatory postsynaptic currents amplitude and surface expression [10]. The apparent discrepancy with our results may be dependent on a number of factors, including different types of stress used, different age of the rats (juvenile versus adult), time points analyzed, and measurement of glutamate receptor subunits expression in different compartments (total membrane fraction versus postsynaptic spine membranes). Moreover, although the changes in AMPA and NMDA receptor subunits expression and phosphorylation levels induced by FS-stress were found to be transient, it cannot be excluded that FS-stress may induce long-lasting alterations of synaptic transmission, mediated by other molecular mechanisms. Further studies are required to address this point.

In previous studies, we showed that FS-stress, together with enhancing depolarization-dependent release of endogenous glutamate, increases excitatory postsynaptic currents amplitude (measured immediately after the stress session) [8]. Acute FS-stress also strongly decreases synaptic facilitation and its calcium-dependence, in line with an increase in release probability. The results obtained in the present study strongly suggest that postsynaptic mechanisms may also be involved in the enhancement of glutamate transmission induced by FS-stress in PFC/FC. In addition, we have also shown recently, by using electron microscopy stereology, that the total number of nonperforated and axoshaft excitatory synapses in medial PFC is increased remarkably (over 40%) immediately after acute FS-stress [38], demonstrating that the early functional changes in glutamate transmission are accompanied by large-scale changes in brain architecture at a fast pace.

## 5. Conclusion

In this study, we reported a time-dependent modulation of both AMPA and NMDA receptor subunits expression and phosphorylation induced by acute FS-stress in PFC/FC.

Although further studies are warranted to dissect the time-dependent functional, molecular, and structural alterations induced by stress in PFC/FC, the present study may further support the evidence of an enhancement of glutamatergic synaptic transmission as early response to acute stress.

## Supplementary Material

The Supplementary Material reports the methods and the results regarding real time PCR and RNA editing analysis of AMPA and NMDA receptor transcripts.

## Figures and Tables

**Table 1:** Corticosterone serum levels.

	*t* = 0	2 h	24 h
Control	60.32 ± 13.06 (*n* = 10)	11.85 ± 3.82 (*n* = 11)	21.53 ± 6.93 (*n* = 9)
FS-stress	308.30 ± 23.30^*∗∗∗*^ (*n* = 10)	57.20 ± 17.34^*∗∗∗*^ (*n* = 11)	53.63 ± 13.41 (*n* = 9)

Data are expressed as ng/mL and reported as mean ± SE. ^*∗∗∗*^ *p* < 0.001.
