# Supplementary material for: Acute Footshock Stress Induces Time-Dependent Modifications of AMPA/NMDA Protein Expression and AMPA Phosphorylation
Source: Neural Plast. 2016 Feb 4;2016:7267865. doi: 10.1155/2016/7267865 (PMC4757710; doi:10.1155/2016/7267865)
Supplement: Supplementary file 1 — The Supplementary Material reports the methods and the results regarding real time PCR and RNA editing analysis of AMPA and NMDA receptor transcripts. [file 7267865.f1.pdf]

**Supplemental Figure 1**

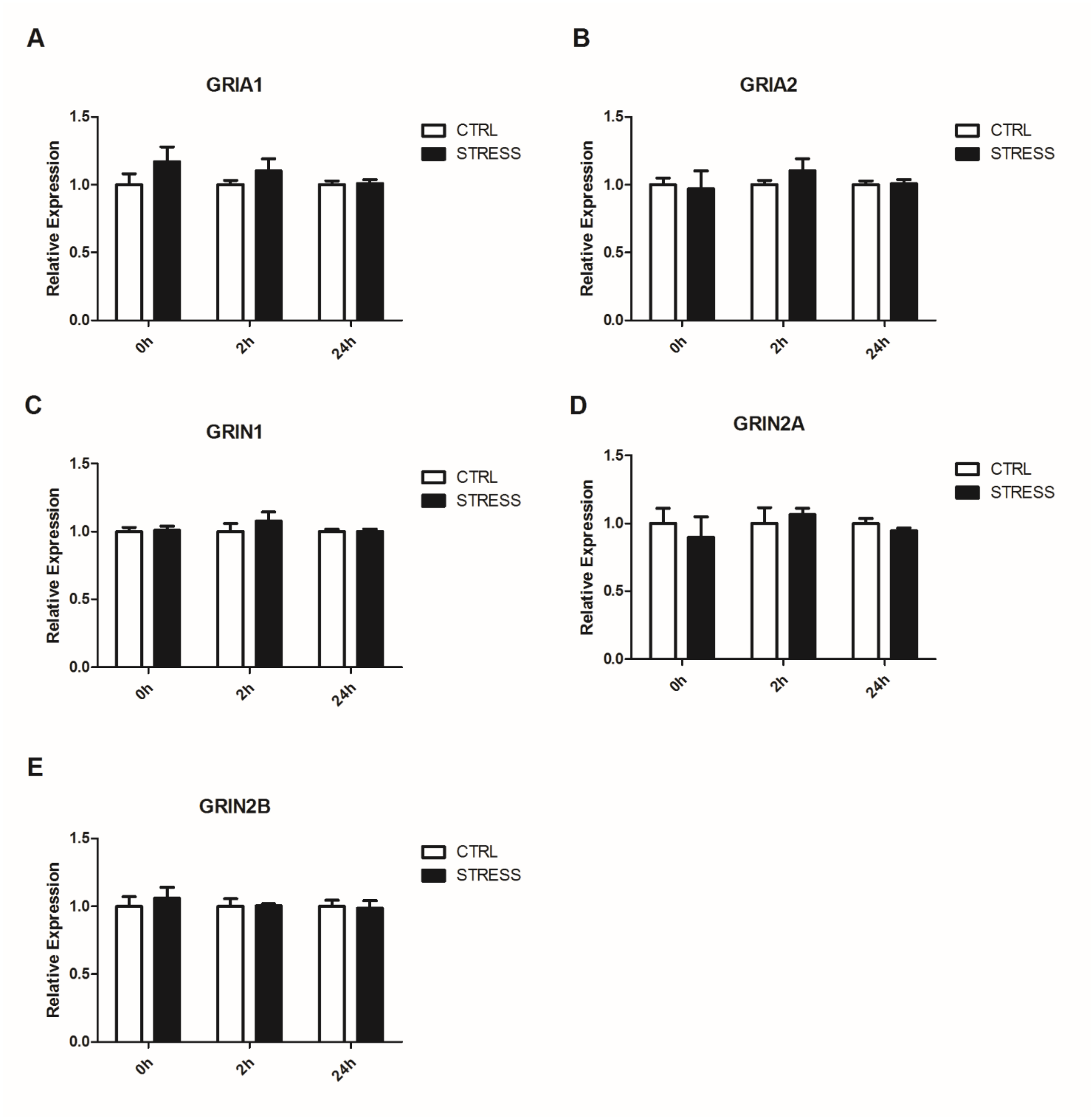


**Supplemental Figure 1.** Expression levels of GRIA1, GRIA2, GRIN1, GRIN2A, GRIN2B mRNA by qPCR. Data are reported as $2^{-\Delta\Delta ct}$ (expression level of control sample is equal to 1) and represent mean values and standard errors obtained from at least three independent evaluations.

**Supplemental Figure 2**

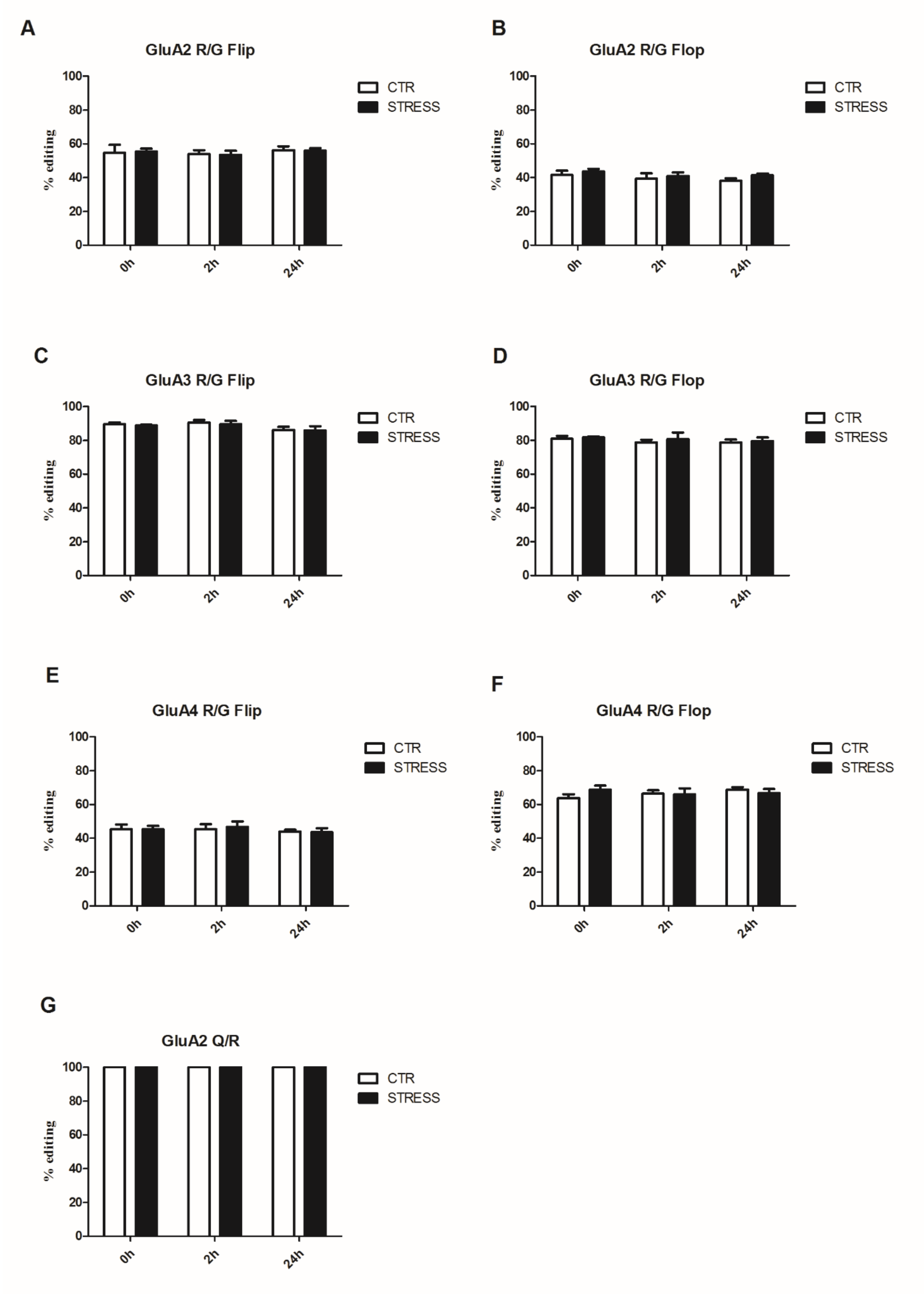


**Supplemental Figure 2:** Editing levels of AMPA receptor editing sites after acute stress.
